# Factors associated with low cure rate of tuberculosis in remote poor areas of Shaanxi Province, China: a case control study

**DOI:** 10.1186/1471-2458-10-112

**Published:** 2010-03-07

**Authors:** Xianqin Ai, Ke Men, Liujia Guo, Tianhua Zhang, Yan Zhao, Xiaolu Sun, Hongwei Zhang, Guangxue He, Marieke J van der Werf, Susan van den Hof

**Affiliations:** 1 Shaanxi Provincial Institute for TB Control and Prevention, Xi'an, Shaanxi province, PR China; 2 The Department of Epidemiology, The Fourth Military Medical University, Xi'an, Shaanxi province, China; 3 Tuberculosis Prevention and Control Center, China CDC, Beijing, China; 4 KNCV Tuberculosis Foundation, The Hague, The Netherlands; 5 Center for Infection and Immunity Amsterdam (CINIMA), University of Amsterdam, Amsterdam, The Netherlands

## Abstract

**Background:**

The directly observed therapy-short course (DOTS) strategy was introduced in Shaanxi province, China to improve tuberculosis (TB) control by means of improved case detection (target: > = 70%) and treatment success rates (target: > = 85%) in new smear positive (SS+) TB patients. At a provincial level the targets were both reached in 2005. However in 30 (28%) out of 107 counties of Shaanxi province the cure rate was below 85%. This study aimed to investigate patient and treatment characteristics associated with non-cure after tuberculosis (TB) treatment in these counties.

**Methods:**

In this case-control study, new smear positive TB cases in 30 counties with a cure rate <85% were included. Cured patients were compared to non-cured patients using logistic regression analysis to assess determinants for non-cure.

**Results:**

Of the 659 patients included, 153 (23.2%) did not have cure as treatment outcome. Interruption of treatment was most strongly associated with non-cure (OR = 8.7, 95% CI 3.9-18.4). Other independent risk factors were co-morbidity, low education level, lack of appetite as an initial symptom of TB disease, diagnosis of TB outside of the government TB control institutes, missing sputum re-examinations during treatment, and not having a treatment observer. Twenty-six percent of patients did not have a treatment observer. The non-cure rate was better for those with a doctor (odds ratio (OR) 0.38, 95% confidence interval (CI) 0.17-0.88) as treatment observer than for those with a family member (OR 0.62, 95%CI 0.37-1.03). The main reason for interrupted treatment mentioned by patients was presence of adverse effects during treatment (46.5%).

**Conclusions:**

Interruption of treatment was most strongly associated with non-cure. Although treatment observation by medical staff is preferred, in order to diminish the proportion of patients who do not have a treatment observer and thereby reduce the proportion of patients who interrupt treatment, we suggest making it possible for family members, after sufficient training, to be treatment observers in remote areas where it is logistically difficult to have village doctors observe treatment for all patients.

## Background

Shaanxi Province is located in the western part of China, covering 20.6 thousand square kilometers with a population of 36.7 million. It is one of the less developed areas in China. The notification rate for new sputum smear positive (SS+) tuberculosis (TB) cases was 29 per 100,000 in Shaanxi province in 2005. The Directly Observed Treatment Short-course (DOTS) strategy, aiming at high-quality TB control, has been implemented in Shaanxi from 2002 onward. Two of the basic components of the DOTS strategy are to realize a high detection rate and a high treatment success rate, in order to control the TB epidemic. Full DOTS coverage in Shaanxi was achieved in 2005. At that time, the SS+ case detection rate in Shaanxi was estimated to be 88%, and the overall cure rate for new SS+ TB cases was also 88% [1]. However, the cure rate of new smear positive patients in 30 (28%) out of the 107 counties of Shaanxi province was below 85%. Most of these counties are located in poor and remote areas of the province.

Several reasons and risk factors for poor TB treatment outcomes have been reported. High age, male sex, low income, no or limited access to transport, distance from home to the treatment centre, incomplete treatment compliance, limited interest in information about the disease and its treatment, limited social support, multidrug resistance and diabetes mellitus have all been found to be related to unsuccessful treatment outcomes [1-7]. It is not clear which factors are major contributors to non-cure of TB patients in the remote and poor areas of Shaanxi Province. This study aims to provide insight into determinants for non-cure among new smear-positive TB patients in the 30 counties in Shaanxi province that did not achieve a 85% cure rate in 2005 despite implementation of the DOTS strategy.

## Methods

### Study population and data collection

New smear positive TB cases who were registered at the TB clinic in the Center for Diseases Prevention and Control (CDC) in 30 counties with low cure rate (<85%) in 2005 from June 1 2006 to March 31 2007 were eligible for participation in this study. A random sample of 540 patients was drawn for inclusion. Patients were interviewed 6-9 months after registration at the CDC. They had all started standardized treatment (2H_3_R_3_Z_3_E_3_/4H_3_R_3_) directly after registration. Information on demographic status, TB disease onset, co-morbidity, the treatment period, as well as on patients' knowledge on TB was collected from the patients by means of standardized questionnaires. Patients are supposed to come for sputum re-examinations at 2, 5, and 6 months after the start of treatment. Information on sputum re-examinations and treatment outcome was obtained from the patient records. Cure was defined according to the international classification [8], i.e. clinical response and two subsequent sputum acid-fast bacilli (AFB) smear negative results, the last one at completion of treatment.

The investigators used standardized questionnaires to interview the patients during scheduled home visits. Assistants rechecked the data after the interviews to ensure completeness.

### Sampling

Sample size calculations were performed using Stat Calc software in Epi-Info 6. A sample size of 540 patients was required to be able to detect a difference of 15% (50% vs. 65%) in a risk factor between two groups with 95% confidence and 80% power, and assuming a rate of loss to follow-up of 10% (e.g. non-participation and incomplete questionnaires).

During the study period, we observed an increased cure rate in the study population compared to 2005. To raise the power of the study to detect risk factors for non-cure, we decided to additionally include all remaining uncured patients registered from June 1 2006 to March 31 2007 that were not sampled already. To avoid information bias, the interviewers were not informed that the additionally included patients all were non-cured.

### Data analysis

Data were double entered in Epi data 3.0, checked for consistency and mistakes were corrected. Statistical analysis was performed in SPSS 13.0. Cured patients were compared to non-cured patients to assess characteristics associated with non-cure. Variables with a p-value < 0.2 in a univariate logistic regression model were included in a multivariate model. The final model was obtained by backward selection of the most parsimonious model based on the log likelihood ratio test (p < 0.05).

### Ethical considerations

The principles of the Helsinki Declaration were taken into account. Written, informed consent was obtained from all study subjects. The study was approved by the medical ethical committee of the Chinese TB Control Association.

## Results

### Characteristics of the patients

During the study period, 659 new smear positive pulmonary TB cases were included, including 45 non-cured patients added later. All patients sampled could be traced and were interviewed at their homes after making an appointment except twenty patients that had died since registration.

Of these 659 patients, 153 (23.2%) were not cured. Thirty-seven (5.6%) completed treatment but did not submit a sputum sample for smear examination at the end of treatment, 82 (12.4%) defaulted from treatment for which 65 had a known reason such as side-effects, 20 (3.0%) died, 9 (1.4%) failed treatment, and 5 (0.8%) transferred out. As it was not possible to obtain reliable information on the twenty patients that had died since registration, they were excluded from the analyses.

Most patients were male (67.3%) and were farmers (62.6%), (Table 1). Of all cases, 39.3% did not have medical insurance which means that they needed to pay for additional medical costs, e.g. for treatment of side-effects, besides smear-examinations and anti-TB drugs which are provided free of charge. Seven percent of the patients had co-morbidity like cardio-vascular diseases, chronic lung disease, or diabetes. Almost half of all patients (43.5%) reported side effects during TB treatment, such as renal disorder (1.4%), jaundice (1.8%), decline in auditory function (5.0%), liver problems (5.3%), joint pain and/or swelling (6.0%), skin rash (11.0%) and so on. In total 46 (7.2%) patients interrupted treatment for a median of 17.5 days before continuing. Twenty-six percent of patients did not have a treatment observer.

**Table 1:** Patient related characteristics associated with non-cure after anti-tuberculosis treatment in patients diagnosed in 30 remote and poor counties in Shaanxi province, China

Patient characteristic	n(%)	Treatment result		OR	95% CI	p-value
					
		Uncured (%)	Cured (%)			
**Sex**						0.92
Female	209 (32.7)	43 (20.6)	166 (79.4)	1		
Male	430 (67.3)	90 (20.9)	340 (79.1)	1.02	0.68-1.54	
**Age (years)**						< 0.01
≤25	141 (22.1)	26 (18.4)	115 (81.6)	1		
26-50	251 (39.3)	38 (15.1)	213 (84.9)	0.79	0.46-1.37	
51-60	105 (16.4)	26 (24.8)	79 (75.2)	1.46	0.79-2.69	
>60	142 (22.2)	43 (30.3)	99 (69.7)	1.92	1.10-3.35	
**Education level**						< 0.01
Illiterate	95 (14.9)	32 (33.7)	63 (66.3)	1		
Primary school	147 (23.1)	31 (21.1)	116 (78.9)	0.53	0.29-0.94	
Junior high school	265 (41.6)	38 (14.3)	227 (85.7)	0.33	0.19-0.57	
Senior high school	106 (16.6)	22 (20.8)	84 (79.2)	0.52	0.27-0.97	
College and above	24 (3.8)	10 (41.7)	14 (58.3)	1.41	0.56-3.52	
**Occupation**						0.04
Farmer	400 (62.6)	77 (19.3)	323 (80.7)	1		
Factory worker	98 (15.3)	16 (16.3)	82 (83.7)	0.82	0.45-1.48	
Other profession worker	141 (22.1)	40 (28.4)	101 (71.6)	1.66	1.07-2.59	
Annual income (CNY)*						0.01
≤1000	187 (29.3)	38 (20.3)	149 (79.7)	1		
1001-2000	195 (30.5)	28 (14.4)	167 (85.6)	0.66	0.39-1.12	
>2000	257 (40.2)	67 (26.1)	190 (73.9)	1.38	0.88-2.17	
**Medical insurance**						0.52
No (own expense)	247 (39.3)	49 (19.8)	198 (80.2)	1		
Yes	382 (60.7)	84 (22.0)	298 (78.0)	1.14	0.77-1.69	
**Distance from patient's home to county TB dispensary**						< 0.01
≤5 km	461(72.1)	81(17.6)	380(82.4)	1		
>5 km	178(27.9)	52(29.2)	126(70.8)	1.93	1.29-2.89	

* Currency rate: US$ 100 equaled about 780 CNY during the study.

### Univariate analysis of risk factors for non-cure

In the univariate analysis, age over 60 years, being illiterate, not having a job, fever and loss of appetite as initial symptoms, diagnosis of TB outside the CDC, co-morbidity, not having a treatment observer, missing more doses of the TB drugs, interrupted treatment, having side effects during treatment, long distance from the patients house to the medical center and having no or irregular sputum examinations were statistically significantly associated with a lower cure rate (Table 1 and Table 2). The population over 60 years had a significantly higher prevalence of co-morbidity (11.4% vs. 5.7%). Also, in aged patients side effects were more frequent (54.2% vs. 42.1%), (Table 3).

**Table 2:** Treatment related characteristics associated with non-cure after anti-tuberculosis treatment in patients diagnosed in 30 remote and poor counties in Shaanxi province, China

Treatment characteristic	n(%)	Treatment result		OR	95% CI	p-value
					
		Uncured (%)	Cured (%)			
**Co-morbidity**						< 0.01
No	562 (93.0)	95 (16.9)	467 (83.1)	1		
Yes	42 (7.0)	22 (52.4)	20 (47.6)	5.41	2.84-10.30	
**Fever**						0.04
No	426 (66.7)	99(23.9)	327 (76.1)	1		
Yes	213 (33.3)	34 (16.0)	179 (84.0)	1.59	1.04-2.45	
**Loss of appetite**						< 0.01
No	497 (77.8)	92 (18.5)	405 (81.5)			
Yes	142 (22.2)	41 (28.9)	101 (71.1)	1.79	1.17-2.74	
**Institute where TB was diagnosed**						0.01
CDC	142 (22.2)	60 (17.1)	290 (82.9)	1		
Other	497 (77.8)	73 (25.3)	216 (74.7)	1.63	1.11-2.40	
**DOT supervisor**						< 0.01
No supervisor	169 (26.4)	48 (28.4)	121 (71.6)	1		
Family	367 (57.4)	73 (19.9)	294 (80.1)	0.63	0.41-0.95	
Doctor	103 (16.1)	12 (11.7)	91 (88.3)	0.33	0.17-0.66	
**Number of times forgotten to take drugs**						< 0.01
none	541 (84.7)	104 (19.2)	437 (80.8)	1		
≤9 times	76 (11.9)	18 (23.7)	58 (76.3)	1.30	0.74-2.31	
>9 times	22 (3.4)	11 (50.0)	11 (50.0)	4.20	1.77-9.96	
**Maximum number of subsequently missed doses**						0.01
None	542 (84.8)	105 (19.4)	437 (80.6)	1		
≤ 3 times	64 (10.0)	14 (21.9)	50 (78.1)	1.17	0.62-2.19	
>3 times	33 (5.2)	14 (42.4)	19 (57.6)	3.07	1.49-6.32	
**Interruption of treament**						< 0.01
No	591 (92.5)	102 (17.3)	489 (82.7)	1		
Yes	48 (7.5)	31 (64.6)	17 (35.4)	8.74	4.66-16.40	
Severity of side effects*						< 0.01
None	361 (56.5)	66 (18.3)	295 (81.7)	1		
Severe	54 (8.5)	27 (50.0)	27 (50.0)	4.47	2.46-8.12	
Medium	41 (6.4)	14 (34.1)	27 (65.9)	2.32	1.15-4.66	
Light	183 (28.6)	26 (14.2)	157 (85.8)	0.74	0.45-1.21	
Sputum re-examination**						< 0.01
Regular	442 (69.2)	57 (12.9)	385 (87.1)	1		
Irregular	155 (24.3)	59 (38.1)	96 (61.9)	4.15	2.71-6.36	
None	42 (6.6)	17 (40.5)	25 (59.5)	4.59	2.34-9.03	

* The severity of side effects is based on the patient's opinion.

** Regular sputum re-examination: New smear positive cases performing sputum re-examination at the end of the 2nd, 5th and 6th month of the therapy, according to the guidelines.

**Table 3:** Association between age and co-morbidityand adverse events in TB patients in Shaanxi province, China

		Age				
	**n**	**>60 year (%)**	**≤60 year (%)**	**OR**	**95% CI**	**p-value**
**Co-morbidity**						0.02
No	562	117(20.8)	445(79.2)	1		
Yes	42	15(35.7)	27(64.3)	2.11	1.09-4.10	
**Adverse events**						0.01
No	353	65(18.4)	288(81.6)	1		
Yes	286	77(26.9)	209(73.1)	1.63	1.10-2.42	

### Multivariate analysis of risk factors for non-cure

Independent risk factors associated with non-cure were being illiterate, having an intermediate level of expenditure, being diagnosed with TB outside the CDC, loss of appetite being an initial symptom of TB, co-morbidity, not having a treatment observer (compared to having a doctor as observer), interruption of treatment, and not or only having irregular sputum re-examinations in the CDC (Table 4).

**Table 4:** Multivariate analysis on risk factors for non-cure after anti-tuberculosis treatment in patients diagnosed in 30 remote and poor counties in Shaanxi province, China

Characteristic	OR	95% CI
**Education level**		
Illiterate	1	
Primary school	0.50	0.24-1.02
Junior high school	0.34	0.18-0.67
Senior high school	0.38	0.17-0.84
College and above	1.67	0.56-4.94
**Annual income (CNY)**		
≤1000	1	
1001-2000	0.71	0.37-1.37
>2000	1.51	0.85-2.68
**Co-morbidity**		
No	1	
Yes	5.80	2.77-12.15
**Loss of appetite**		
No	1	
Yes	1.77	1.03-3.06
**Institute where TB was diagnosed**		
CDC	1	
Other	1.79	1.10-2.92
**DOT supervisor**		
No supervisor	1	
Family	0.62	0.37-1.03
Doctor	0.38	0.17-0.88
**Interruption of treatment**		
No	1	
Yes	8.41	3.85-18.37
**Sputum re-examinations**		
Regular	1	
Irregular	2.56	1.52-4.32
No	2.10	0.90-4.91

*Currency rate: US $100 equals about 780 CNY during the study.

Although being a farmer, high age, having fever as an initial symptom and presence of side-effects during treatment were statistically significant risk factors for non-cure in the multivariate model, removal of these variables did not significantly reduce the fit of the model as tested with the log likelihood ratio test, so we excluded them from the final model.

**Figure 1:**
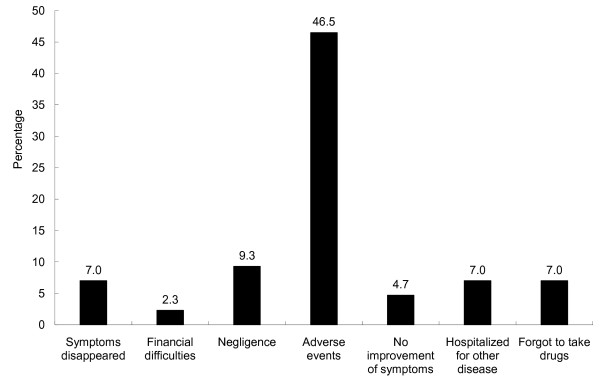
**Reasons for interruption of treatment as reported by patients (n = 43)**. Patients could provide more than one reason.

Interruption of treatment was most strongly associated with non-cure (OR = 8.7, 95% CI 3.9-18.4). Having side effects was the main reason for interrupting treatment (46.5%, Figure 1).

**Figure 2:**
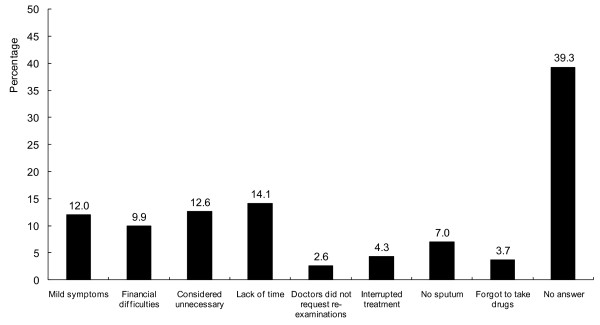
**Reasons for the absence and irregular sputum re-examination as reported by patients (n = 187)**. Patients could provide more than one reason.

Several reasons were mentioned by patients for not going to the clinic for (regular) sputum re-examination. The most common reasons mentioned were: having mild symptoms, not thinking it was necessary to have regular re-examinations, and financial or traffic difficulties (Figure 2). Thirty-nine percent of the respondents did not provide a reason.

## Discussion

It is assumed that by increasing the detection rate and cure rate to at least 70% and 85% respectively, transmission of TB and morbidity and mortality of TB disease will be reduced, and the TB epidemic would be controlled effectively [9,10]. The case detection rate in Shaanxi province in China is assumed to be sufficient, but the cure rate did not reach the target of 85% in all counties in the province.

Many factors were observed to be related to non-cure in this study, including patient, diagnosis and treatment related factors. Independent risk factors for non-cure of new smear-positive TB patients in the selected counties with a low cure rate in Shaanxi province were: presence of co-morbidity, loss of appetite as an initial symptom of TB, diagnosis outside the government TB control institute, failing to perform regular sputum re-examinations, temporary interruption of treatment, and no direct observation of treatment by medical staff.

Among the independent risk factors for non-cure, interruption of treatment was the most important one (OR = 8.7). The results are supported by results from other studies [2,8,11]. One study from China showed that there were different reasons for treatment interruption such as side-effects and financial difficulties in relation to treatment [12]. Patients who interrupt treatment are more likely to become infectious again and acquire drug resistance [13,14]. Thus, improvement of adherence to treatment is important to control (drug-resistant) TB.

Co-morbidity was the other major predictor for non-cure in our study population (OR = 5.8). Our result showed that the aged population had a higher prevalence of co-morbidity and side effects, as shown before [15]. So there is a correlation between high age, co-morbidity and presence of side-effects. Although co-morbidity, high age and side-effects were all significantly associated with non-cure, co-morbidity turned out to be the most important predictor for non-cure in our study. The pre-existence of other diseases, especially diabetes mellitus, has been shown to be associated with treatment failure before [6]. Older age has been associated with unsuccessful treatment outcome before, although not consistently [2-4,7].

Hernandez-Garduno and Perez-Guzman hypothesized that chronic lack of appetite can be a potential independent risk factor for TB disease and can affect TB treatment [16]. Our result confirmed that a lack of appetite as a symptom of TB is risk factor for poor treatment outcomes.

Not having the standard sputum re-examinations was associated with non cure. The reasons given by patients for missing re-examinations, indicated that many patients may not have been aware of the importance of re-examination.

Patients with village doctors as treatment observer had a decreased risk of non-cure compared to patients without treatment observer. Patients with observed by family members also had a decreased risk of non-cure (p = 0.07), although the risk was slightly higher than for those patients observed by village doctors. Daniel et al. studied the risk factors associated with default from tuberculosis treatment in Nigeria and also suggested that alternative strategies such as the use of family members to oversee treatment may be desirable [4]. Our results support this point.

Contradictory results exist on the effect of poverty and male sex on treatment outcomes [4,7]. In our study these were not found to be independent risk factors. Individual immune status [17], multidrug resistance [18,19], and the *Mycobacterium tuberculosis *Beijing genotype have been associated with treatment failure [20], but we were unable to study these factors in our study.

A limitation of our study is that we mostly had to rely on self-reported status of sensitive issues like interruption of treatment, and had to ask retrospectively about treatment issues like number of missed doses and side-effects. Also, although we did not inform interviewers of treatment outcomes, it is possible that they did find out during the interviews. By means of careful training of interviewers and interviewing with fixed questions we feel we were able to obtain quite reliable answers. During data collection we decided to additionally include all remaining non-cured cases in the study population as cure rates were higher than in the previous year. Exclusion of the 45 non-cured cases added later to the study sample led to similar results with the same major risk factors as co-morbidity, treatment observation, TB diagnosis institute and attendance to sputum smear examinations still included in the final multivariate model.

Our study has provided us with useful insights on factors influencing non-cure in the poor and remote areas of Shaanxi province. Based on the results, we recommend that patient education should be enhanced by clinic doctors to improve the patients understanding of their disease and its treatment and to improve compliance to treatment and re-examinations. Furthermore, during treatment, more attention should be paid to monitoring of side-effects. Limiting fees for treatment of side effects may reduce interruption of treatment and increase cure rates. Treatment observation of patients, including observation by the county and village doctors should be enhanced. Because of the poor transportation possibilities to reach the TB clinics and wide dispersal of TB patients, we suggest that DOT supervision in remote areas can be performed by family members after they are trained to ensure quality of treatment observation.

## Conclusions

Interruption of treatment and co-morbidity were the most important predictors of non-cure. Appropriate patient education and support, and treatment of co-existing diseases may increase compliance, prevent interruption of treatment and hereby increase the cure rate. Twenty-six percent of patients did not have a treatment observer. Although treatment observation by medical staff is preferred, in remote areas where it is logistically difficult to have village doctors observe treatment of all patients, we suggest making it possible for family members to become treatment observers after they are properly trained.

## Competing interests

The authors declare that they have no competing interests.

## Authors' contributions

XA and KM designed the protocol, carried out the field work, did data analysis and drafted the manuscript. LG and TZ participated in the write up of the protocol. YZ, XS, HZ carried out the field work, participated in the data analysis and write up of the manuscript. GH, MW and SH participated in the write up of the protocol, in the data analysis and write up of the manuscript. All authors read and approved the final manuscript.

## Pre-publication history

The pre-publication history for this paper can be accessed here:

http://www.biomedcentral.com/1471-2458/10/112/prepub
